# Supplementary material for: Bifidobacterium animalis A12, a Probiotic Strain That Promotes Glucose and Lipid Metabolism, Improved the Texture and Aroma of the Fermented Sausage
Source: Foods. 2023 Jan 10;12(2):336. doi: 10.3390/foods12020336 (PMC9858581; doi:10.3390/foods12020336)
Supplement: Supplementary file 1 [file foods-12-00336-s001.zip › Supplementary materials.pdf]

Table S1 Content of volatile flavour components in *B. animalis* A12 fermented sausage

**Volatile compounds**	Content（μg/kg）	
	CK	A12
**Aldehydes**	783.69±57.31[b]	986.29±104.77[a]
N-octanal	51.17±3.36[b]	83.62±5.57[a]
Nonanal	60.02±4.35[b]	217.62±14.36[a]
Dodecanal	330.15±12.17[a]	31.37±2.25[b]
Hexanal	—	36.21±3.95[a]
Heptanal	—	63.73±6.54[a]
Benzaldehyde	72.13±5.57[a]	68.42±6.63[a]
Tetradecanal	107.43±10.23[a]	17.56±1.23[b]
Trans-2-octenal	—	21.53±1.11[a]
Decylaldehyde	—	30.52±2.74[a]
Trans-2-nonanal	—	37.61±3.51[a]
Cis-2-decanal	—	114.11±13.79[a]
Cis-2-heptanal	—	45.71±15.66[a]
Isovaleraldehyde	—	8.77±1.31[a]
4 - methoxy-benzaldehyde	—	21.98±2.45[a]
Hexadecanal	162.79±21.63[a]	157.53±23.67[a]
**Alcohols**	233.62±32.12[a]	105.42±10.50[b]
1-octen-3-ol	—	20.77±1.54[a]
Furfuryl alcohol	—	8.41±0.31[a]
N-hexanol	—	12.25±1.05[a]
Phenylethyl alcohol	—	15.78±1.32[a]
N-octanol	—	36.42±5.33[a]
1-nonanol	—	1.06±0.03[a]
Undecyl alcohol	—	10.73±0.92[a]
1 - Heptantriol	233.62±32.12[a]	—
**Ketones**	72.93±9.73[a]	73.15±5.56[a]
2-pentadecanone	72.93±9.73[a]	11.81±0.78[b]
2-nonanone	—	5.25±0.12[a]
Hydroxyacetone	—	28.31±2.25[a]
Acetoin	—	27.78±2.41[a]
**Acids**	318.48±30.82[a]	251.20±23.72[b]
N-nonanoic acid	94.05±6.67[a]	99.20±8.47[a]
Acetic acid	—	105.44±8.77[a]
Capric acid	84.03±7.64[a]	—
N-caproic acid	—	43.42±6.33[a]
Lauric acid	140.40±16.51[a]	—
3 - methylthiophene aldehyde	—	3.14±0.15[a]
**Esters**	111.65±8.87[a]	28.58±2.87[b]
Ethyl ocanoate	—	1.41±0.15[a]
2 - hexyl-1,1 - dicyclopropane-2 - octanoic acid methyl ester	—	6.22±0.73[a]

Dibutylphthalate	111.65±8.87a	14.50±1.22b
α, β-Glycerol dipalmitate	—	1.42±0.17a
Ethylene hexanoate	—	1.47±0.05a
Benzyl oleate	—	2.27±0.44a
Furfuryl acetate	—	8.92±0.99a
alkenes	—	2.51±0.18a
styrene	—	0.76±0.03a
3 - Ethyl-2 - methyl-1,3 - hexadiene	—	1.75±0.15a
**other**	209.46±31.97a	139.54±17.10b
2-Pentylfuran	27.99±1.66b	104.53±14.11a
2,6-Ditert-butyl hydroquinone	7.20±0.37b	12.33±1.13a
2,4-di-tert-butylphenol	—	4.12±0.27a
3-cyclohexene-1-nitrile	—	5.57±0.45a
1-Nitrohexane	—	2.38±0.27a
N-nonadecane	—	6.69±0.69a
Tetradecane	63.30±10.21a	3.92±0.22b
Octadecane	110.97±19.73a	—
**Total**	1729.83±170.82a	1586.69±164.70a

a, b, c means in the same row with different letters are significantly different (*P<0.05*).

Table S2 Content of hydrolyzed amino acids in experimental group sausage fermented at different time (mg / kg dry matter)

	0h	6h	12h	18h	24h
Thr	28.48±3.65b	28.32±2.26b	32.35±4.85a	33.18±4.77a	30.08±5.15b
Val	60.40±4.21b	59.33±3.79b	70.17±6.78a	75.46±7.32a	64.81±5.12b
Met	6.87±0.44b	7.35±0.33a	6.17±0.25c	7.18±0.64ab	5.99±0.33c
Ile	40.98±4.67c	44.73±5.33c	53.02±3.51a	56.24±2.47a	48.63±3.11ab
Leu	81.13±16.31b	88.68±13.49b	106.01±12.37a	113.18±11.25a	101.96±15.47a
Phe	48.50±2.33c	50.31±2.74c	60.08±4.41a	64.48±3.37a	55.39±3.64b
Lys	93.59±13.39b	94.47±12.48b	115.83±15.29a	119.48±14.27a	106.62±9.87a
Asp	77.26±7.88b	79.97±8.19b	91.60±8.66a	96.22±7.98a	84.45±7.35b
Glu	40.37±7.92c	52.75±5.44b	61.02±4.48a	64.94±5.19a	55.16±6.12b
Gly	50.32±4.67b	52.26±5.13b	52.78±3.88b	63.16±7.21a	51.28±4.32b
Ser	119.17±19.85b	124.57±23.59b	147.07±19.87a	157.69±21.51a	134.99±22.37b
Ala	57.67±5.12c	56.18±3.67c	66.76±4.82b	71.28±3.57a	61.10±5.19b
Cys	4.15±1.15a	4.25±1.35a	4.58±0.98a	5.03±1.02a	4.57±1.03a
Tyr	29.97±3.37b	28.63±3.61b	33.95±2.98a	36.44±2.75a	32.90±3.31a
His	35.56±2.61c	36.15±2.57c	43.97±3.43b	51.69±4.97a	46.69±3.65b
EAA	359.96±45.00b	373.18±40.42b	443.62±47.46a	469.20±44.09a	413.48±42.69a
NAA	422.478±52.57c	434.77±53.55c	501.74±49.10b	546.44±54.20a	471.15±53.34b
E / N ratio	0.852	0.858	0.884	0.859	0.878
Total	782.43±97.57b	807.95±93.97b	945.36±96.56a	1015.63±98.29a	884.63±96.03b

EAA: essential amino acids; NAA: nonessential amino acid;

a, b, c means in the same row with different letters are significantly different ($P<0.05$).

Table S3 Results of volatile flavour compounds in fermented experimental group sausage by GC-MS analysis

**Volatile compounds**	Content（μg/kg）				
	0h	6h	12h	18h	24h
**Aldehydes**	95.23± 10.13[d]	160.89± 8.24[c]	340.30± 22.42[b]	416.56± 26.19[a]	284.76± 19.31[b]
N-octanal	23.66± 2.36[d]	33.35± 1.98[c]	33.29± 3.13[c]	90.71± 3.13[a]	47.23± 1.74[b]
Nonanal	60.23± 3.39[c]	40.29± 5.51[d]	129.12± 6.61[a]	69.73± 5.56[b]	70.63± 4.22[b]
Dodecanal	11.34± 0.73[a]	2.23± 0.44[d]	4.03± 0.22[b]	2.79± 0.13[c]	2.31± 0.57[d]
Hexanal	—	6.65± 0.31[c]	21.48± 2.27[a]	20.25± 2.15a[b]	15.54± 1.38[b]
Heptanal	—	7.35± 0.82[c]	13.11± 0.48[b]	23.72± 1.05[a]	13.53± 0.46[b]
Benzaldehyde	—	15.63± 1.32[d]	22.46± 1.43[b]	37.21± 2.53[a]	19.49± 1.73[c]
Tetradecanal	—	17.63± 0.47[b]	16.94± 0.22[b]	34.93± 3.49[a]	1.50± 0.26[c]
Trans-2-octenal	—	3.51± 0.23[c]	6.07± 0.75[b]	11.75± 1.68[a]	6.12± 0.25[b]
Decylaldehyde	—	3.93± 0.28[c]	4.11± 0.17[c]	12.55± 1.35[a]	7.78± 0.67[b]
Trans-2 - nonanal	—	6.29± 0.45[c]	13.84± 1.53[b]	22.24± 1.27[a]	13.08± 1.69[b]
Cis-2 - decanal	—	11.64± 1.58[d]	14.80± 1.40[c]	44.24± 1.45[a]	27.00± 1.25[b]
Cis-2 - heptanal	—	4.28± 0.39[d]	7.27± 0.97[c]	21.74± 0.32[a]	12.54± 0.71[b]
Isovaleraldehyde	—	4.62± 0.49[a]	3.99± 0.26[b]	4.39± 0.37[a]	3.56± 0.27[c]
Cis-2 - heptanal	—	0.49± 0.08[c]	1.45± 0.28[b]	2.76± 0.51[a]	1.56± 0.36[b]
Hexadecanal	21.57± 3.65[c]	—	42.04± 2.08[a]	—	35.53± 2.87[b]
3-Methylthiopropionaldehyde	—	1.49± 0.54[c]	—	9.75± 0.48[a]	2.24± 0.44[b]
2-Bromooctadecaldehyde	—	1.51± 0.08[a]	1.09± 0.07[b]	0.31± 0.05[c]	—
3 - Methylthiophene aldehyde	—	—	1.18± 0.09[a]	—	1.13± 0.07[a]

**Volatile compounds**	Content（μg/kg）				
	0h	6h	12h	18h	24h
Fifteen carbonaldehyde	—	—	1.28±0.12^{b}	1.81±0.13^{a}	1.30±0.08^{b}
13-Cis-octadecylenal	—	—	1.64±0.28^{b}	2.31±0.29^{a}	1.65±0.23^{b}
Undecanal	—	—	—	1.70±0.18^{a}	1.04±0.06^{b}
2,4-Nonadienal	—	—	1.11±0.06^{b}	1.67±0.07^{a}	—
**Alcohols**	—	9.89±1.29^{c}	21.79±2.26^{b}	35.35±1.95^{a}	24.87±2.18^{ab}
1-Octen-3-ol	—	1.54±0.33^{d}	2.27±0.30^{c}	4.96±0.44^{a}	3.81±0.57^{ab}
N-hexanol	—	1.36±0.27^{c}	1.31±0.14^{c}	3.25±0.18^{a}	1.86±0.27^{b}
Trans-2-decenol	—	1.17±0.26^{d}	2.47±0.26^{b}	3.86±0.23^{a}	2.16±0.15^{c}
Phenylethyl alcohol	—	—	4.03±0.75^{b}	5.84±0.72^{a}	3.95±0.52^{b}
Furfurylmercaptan	—	0.66±0.09^{d}	1.17±0.05^{c}	1.57±0.04^{b}	1.83±0.07^{a}
N-octanol	—	4.11±0.28^{d}	7.23±0.39^{b}	11.58±0.21^{a}	6.57±0.24^{c}
Undecyl alcohol	—	1.05±0.06^{c}	0.89±0.02^{b}	2.17±0.05^{a}	—
3-Decayne-2-ol	—	—	—	2.12±0.08^{b}	2.42±0.05^{a}
Ethyl alcohol	—	—	2.42±0.35^{a}	—	2.27±0.31^{a}
**Ketones**	5.35±0.88^{c}	9.55±1.54^{c}	12.20±1.66^{bc}	22.40±1.61^{a}	14.39±1.13^{b}
2-Pentadecanone	2.92±0.52^{b}	1.63±0.51^{d}	2.32±0.46^{c}	3.46±0.44^{a}	1.48±0.43^{d}
2-Nonanone	—	2.81±0.18^{d}	3.91±0.39^{c}	9.32±0.42^{a}	6.96±0.43^{b}
4,5-Dimethyl-1,3-dioxacyclopentene-2-one	—	2.17±0.36^{c}	3.66±0.52^{c}	8.83±0.68^{a}	5.13±0.23^{b}
Methylheptenone	—	0.53±0.04^{b}	0.66±0.03^{ab}	0.79±0.07^{a}	0.82±0.04^{a}
Hydroxyacetone	2.43±0.36^{a}	2.41±0.45^{a}	1.65±0.26^{b}	—	—
**Esters**	4.78±0.51^{c}	9.08±0.70^{b}	13.15±0.86^{a}	14.22±1.34^{a}	11.11±1.01^{a}

Volatile compounds	Content（μg/kg）				
	0h	6h	12h	18h	24h
Ethyl ocanoate	—	1.04±0.07[b]	1.61±0.03[a]	1.61±0.04[a]	1.69±0.07[a]
2-Hexyl-1,1- dicyclopropane-2 -octanoic acid methyl ester	—	0.25±0.04[c]	0.34±0.02[bc]	1.31±0.12[a]	0.64±0.15[b]
Dibutylphthalate	4.78±0.51[a]	3.87±0.21[b]	4.34±0.48[a]	4.58±0.56[a]	3.85±0.28[b]
α, β-Glycerol dipalmitate	—	0.69±0.05[b]	2.01±0.08[a]	0.16±0.02[b]	—
Ethylene hexanoate	—	1.70±0.14[b]	3.00±0.15[a]	3.21±0.21[a]	1.14±0.15[b]
Benzyl oleate	—	—	0.39±0.06[b]	1.30±0.14[a]	1.11±0.18[a]
N-decyl decanoate	—	—	3.41±0.09[a]	2.59±0.23[b]	2.45±0.11[b]
Methyl 12,15-octadecanoate	—	0.86±0.04[a]	—	1.09±0.05[a]	1.12±0.06[a]
γ-butyrolactone	—	1.96±0.26[a]	—	1.29±0.13[b]	1.44±0.23[b]
**Acids**	42.63±8.86[c]	20.10±4.96[d]	88.53±14.00[a]	96.15±15.51[a]	72.45±11.31[b]
N-nonanoic acid	14.05±2.16[a]	1.93±0.62[c]	7.08±1.46[b]	6.73±1.45[b]	6.14±1.83[b]
Acetic acid	—	15.47±3.84[c]	45.35±5.52[a]	21.81±2.02[b]	20.84±1.46[b]
Capric acid	4.03±0.48[a]	0.95±0.25[c]	2.89±0.47[b]	2.78±0.26[b]	2.65±0.27[b]
N-caproic acid	—	—	15.57±3.45[b]	30.68±2.77[a]	23.51±4.19[b]
Caprylic acid	—	—	5.04±0.61[c]	11.22±2.99[a]	7.53±0.92[b]
Lauric acid	9.40±1.45[a]	1.75±0.25[c]	3.44±0.71[b]	2.00±0.22[b]	2.49±0.28[b]
Palmitic acid	15.15±4.77[a]	—	8.02±1.67[b]	17.24±5.13[a]	5.92±1.68[c]
Butyric acid	—	—	1.14±0.11[b]	3.69±0.67[a]	3.37±0.68[a]
**Others**	37.03±11.02[c]	33.47±7.48[c]	48.68±7.27[b]	65.19±8.35[a]	49.83±8.67[b]
2-Pentylfuran	27.98±8.44[c]	27.24±5.76[c]	43.23±5.62[b]	56.19±6.48[a]	46.13±7.51[ab]
N-nonadecane	—	1.64±0.59[a]	1.09±0.63[a]	1.60±0.71[a]	0.74±0.51[a]

**Volatile compounds**	Content（μg/kg）				
	0h	6h	12h	18h	24h
Hexadecane	9.05± 2.58[a]	1.86± 0.38[b]	2.28± 0.54[b]	2.98± 0.51[b]	1.69± 0.37[b]
1,6-Caprolactam	—	2.73± 0.75[b]	2.08± 0.48[bc]	4.42± 0.65[a]	1.27± 0.28[c]
Total	179.67± 30.52[d]	233.43± 22.67[c]	512.45± 46.81[b]	627.47± 53.34[a]	443.02± 42.48[ab]

a, b, c, d: means in the same row with different letters are significantly different (*P<0.05*)
